# Regulatory Effect of 1,25(OH)2D3 on TGF-*β*1 and miR-130b Expression in Streptozotocin-Induced Diabetic Nephropathy in Rats

**DOI:** 10.1155/2019/1231346

**Published:** 2019-11-03

**Authors:** Yuetong Liu, Ye Yang, Qin Wang, Apaer Kahaer, Jiyun Zhang, Jing Liao, Mairemugu Abudureyimu, Reyila Yahefu, Jing Qi, Lei Zhao, Jun Zhu

**Affiliations:** ^1^Department of Endocrinology, The First Affiliated Hospital of Xinjiang Medical University, Urumqi 830054, Xinjiang, China; ^2^Department of No. 1 Cadres, The Second Affiliated Hospital of Xinjiang Medical University, Urumqi 830063, Xinjiang, China; ^3^Department of Rheumatology and Immunology, The Second Affiliated Hospital of Xinjiang Medical University, Urumqi 830063, Xinjiang, China; ^4^Department of Chu Medical, The Second Affiliated Hospital of Xinjiang Medical University, Urumqi 830063, Xinjiang, China; ^5^Department of Endocrinology, People's Hospital of Shenzhen Baoan District, The Second School of Clinical Medicine, Southern Medical University, Shenzhen, Guangdong 518101, China

## Abstract

**Objective:**

To investigate the role of microRNA-130b in 1,25(OH)2D3 mediated improvement of renal fibrosis via transforming growth factor-beta 1 in a rat model of diabetic nephropathy (DN).

**Methods:**

DN was induced in 30 rats by intraperitoneal injection of streptozotocin. These rats were randomly allocated to the DN group, TGF-*β*1 overexpression group (*in situ* injection of TGF-*β*1 lentivirus to kidney tissues), and TGF-*β*1 siRNA group (*in situ* injection of TGF-*β*1 siRNA lentivirus to kidney tissues). Rats with different expression levels of TGF-*β*1 were administered 1,25(OH)2D3 (0.03 *μ*g/kg/d) or peanut oil as control. DN rats were treated only with peanut oil. All rats were randomly divided into five groups (*n* = 6 per group): TGF-*β*1 overexpression + oil, TGF-*β*1 overexpression + 1,25(OH)2D3, TGF-*β*1 siRNA + oil, TGF-*β*1 siRNA + 1,25(OH)2D3, and DN + oil groups. After 37 days, kidney samples were collected and the expression of TGF-*β*1 and miR-130b was determined by real-time PCR, western blotting, and immunohistochemistry. Hematoxylin and eosin staining and Masson staining were used to evaluate kidney morphological and fibrogenic changes. Differences were determined using ANOVA and Student's *t*-test.

**Results:**

RT-PCR, western blotting, and immunohistochemistry revealed that interference of TGF-*β*1 significantly decreased mRNA and protein levels of TGF-*β*1 in renal tissues of DN rats compared to those in renal tissues of rats overexpressing TGF-*β*1 (*p* < 0.05). Histological analysis showed that upregulated TGF-*β*1 led to disorganized kidney structure and severe kidney fibrosis. The expression of miR-130b was significantly lowered upon lentivirus-mediated overexpression of TGF-*β*1 than upon downregulation of TGF-*β*1 (*p* < 0.05). Treatment with 1,25(OH)2D3 led to a significant reduction of TGF-*β*1 at the mRNA and protein levels (both *p* < 0.05), improvement of renal structure and fibrosis, and an increase in miR-130b expression (*p* < 0.05).

**Conclusion:**

TGF-*β*1 can decrease the expression of miR-130b in kidney tissues of DN rats. Moreover, miR-130b may be involved in the protective effect of 1,25(OH)2D3 on renal fibrosis via TGF-*β*1.

## 1. Introduction

Diabetic nephropathy (DN) is the most common complication of diabetes and the main cause of end-stage renal failure disease. Fibrosis is a common pathogenic mechanism of DN and other chronic kidney diseases progressing to end-stage renal disease. During this progression, transforming growth factor-beta 1 (TGF-*β*1) is the most important fibrogenic factor [1–3] involved in promoting the proliferation of fibroblasts and stimulating the deposition of a variety of extracellular matrix (ECM) materials [4]. 1,25-Dihydroxyvitamin D3 (1,25(OH)2D3) can inhibit the renin-angiotensin system, inflammatory responses, and proteinuria. Moreover, it can also alleviate fibrosis in DN by regulating TGF-*β*1 expression [5].

Recently, microRNAs (miRNAs) have become a research hotspot. MiRNAs are a group of highly conserved endogenous noncoding small RNAs that suppress transcription and translation by binding to the 3′-untranslated region (3′-UTR) of the targeted mRNA. Approximately 60% of human genes are regulated by miRNAs [6], which play diverse roles in pathological processes including autophagy, diabetes, and fibrosis [7–9]. In high glucose-treated endothelial cells, the expression levels of senescence-associated secretory phenotype (SASP) miRNA, such as miR-146a and miR-21, were significantly increased, while the expression level of miR-192 was decreased [10]. It has been found that TGF-*β* has a biphasic induction process on miR-192 expression in mouse mesangial cells in which AKT-activated P300-induced transcription factors Ets-1 and histone H3 acetylation ultimately leads to the sustained expression of Smad [11]. The expression level of miRNA-22 is significantly increased during the pathogenesis of diabetic nephropathy, and miRNA-22 promotes the expression of type IV collagen (Col IV) and inhibits the autophagy of renal tubular epithelial cells by targeting phosphatase and tensin homolog (PTEN), inducing tubulointerstitial fibrosis and promoting the development of diabetic nephropathy [12]. Among them, the miR-130 family is associated with the fibrosis of multiple organs. For example, miR-130a inhibits angiotensin II-mediated myocardial fibrosis, and overexpression of miR-130a can improve myocardial function, promote angiogenesis, and reduce collagen deposition after myocardial infarction in mice [13]. Such protective effects of miR-130a may be associated with the inhibition of PTEN (phosphatase and tensin homolog deleted on chromosome 10) and activation of the phosphoinositide 3-kinase/protein kinase B (PI3K/Akt) signaling pathway [14]. Moreover, miR-130a-3p could inhibit the activation of hepatic stellate cells and prevent nonalcoholic hepatic fibrosis [15]. A recent study demonstrated that miR-130b can decrease fat deposition, reverse glucose tolerance, and improve high-fat-induced obesity in C57BL/6 mice [16]. Moreover, it has been shown that miR-130b plays a protective role in the fibrosis of diabetic nephropathy by mediating the cascade amplification induced by TGF-*β*1 stimulation. High expression of TGF-*β*1 leads to down-regulated miR-130b, inducing elevated expression of fibrotic factors including COL4A1 and PAI [17, 18]. In addition, serum miR-130b is predicted to be a novel biomarker for the early diagnosis of type II diabetes [19].

Presently, a rat model of type II DN was established through the injection of streptozotocin (STZ) [20]. Lentivirus-mediated up-or downregulation of TGF-*β*1 was used to investigate the association between miR-130b and TGF-*β*1. After 1,25(OH)2D3 intervention, we explored the involvement of miR-130b in the 1,25(OH)2D3-mediated improvement of renal fibrosis in rats via TGF-*β*1.

## 2. Materials and Methods

### 2.1. Research Design and Methods

All procedures were approved by the Animal Ethic Committee of Animal Experimental Center of Xinjiang Medical University (Xinjiang, China; IACUC-20181020-01). The experiments were completed in the Animal Experimental Center of Xinjiang Medical University and the laboratory of metabolic diseases of the clinical research institute of Xinjiang Medical University (Xinjiang, China).

### 2.2. Reagents

STZ was purchased from Sigma-Aldrich (USA). The RNA stabilization solution was purchased from Ambion (USA). Calcitriol capsules were purchased from Roche Diagnostics (China; J20100056, 0.25 *μ*g). Lentivirus vectors used to overexpress or downregulate TGF-*β*1 were constructed by Jikai Gene Technology Co., Ltd. (China). RNA extraction, cDNA reverse transcription, and real-time quantitative PCR kits were purchased from QIAGEN (Germany). Rabbit antirat TGF-*β*1 monoclonal antibody was purchased from Abcam (UK).

### 2.3. Animals

Forty specific pathogen-free male Sprague-Dawley (SD) rats weighing 180 to 220 g purchased from Cloud-Clone Corp. (China) were fed a high-fat diet for 8 weeks. The rats weighed 400 to 450 g at the end of this period. STZ (30 mg/kg) was intraperitoneally injected, and the high-fat diet was continued. After 3 days, blood was collected from the caudal vein. The diabetic model was considered successful if the blood glucose was ≥16.7 mmol/L [21]. The high-fat diet was continued, and the blood glucose was measured routinely. After 4 weeks, the DN model was considered successful if the 24 h urinary albumin excretion was >20 mg and urine was positive for glucose. Thirty surviving rats were randomly divided into five groups (*n* = 6 per group): TGF-*β*1 overexpression + peanut oil, TGF-*β*1 overexpression + 1,25(OH)2D3, TGF-*β*1 siRNA + peanut oil, TGF-*β*1 siRNA + 1,25(OH)2D3, and DN + peanut oil. Rats were sacrificed after 37 days.

### 2.4. Equipment

A blood glucose monitor device was purchased from ACCU-Chek (Germany). The model CFX96 real-time fluorescent quantitative PCR instrument was purchased from BIO-RAD (USA), as were the gel imager and electrophoresis system. The ultraviolet (UV) spectrophotometer was purchased from Thermo Fisher Scientific (USA).

### 2.5. Sample Collection

After treatment with 1,25(OH)2D3 or peanut oil for 37 days, the rats were anesthetized using ketamine (50 mg/kg). Their kidneys were harvested, washed, and weighed. Some tissues were immersed in RNAlater solution and stored at −80°C [22]. The remaining samples were fixed in 4% paraformaldehyde.

### 2.6. Histological Analysis

Kidney tissues were fixed in 4% paraformaldehyde for 48 h and embedded in paraffin. Sections were prepared for hematoxylin and eosin (HE) staining to observe the morphological changes of glomeruli, renal capsules, and tubules. Masson staining was performed to observe the kidney structure and fibrosis. Blue-stained tissue indicated renal fibrosis, and red-stained tissue suggested normal kidney tissue.

### 2.7. Immunohistochemistry

Kidney tissues were fixed in 4% paraformaldehyde for 48 h and embedded in paraffin. Sections were prepared for immunohistochemistry (IHC) measurement of TGF-*β*1 expression. Phosphate-buffered saline was used as the negative control. TGF-*β*1 positive cells were brownish yellow in color.

### 2.8. Real-Time PCR

Total RNA was extracted from the renal cortex (0.15 g) using TRIzol reagent and reverse transcribed to cDNA. Gene sequences of primers are shown in Table 1. For determination of TGF-*β*1 expression, real-time PCR was performed by 40 cycles of denaturing at 95°C for 10 min, annealing at 60°C for 1 min, and extension at 95°C for 15 s. *β*-Actin was used as the reference. For determination of miR-130b, PCR reaction was performed by 40 cycles of denaturing at 94°C for 15 min, annealing at 55°C for 30 s, and extension at 70°C for 30 s. has-U6 was selected as the reference. All experiments were performed in triplicates, and mRNA expression of TGF-*β*1 and miR-130b was determined using the 2^−△△Ct^ method.

### 2.9. Western Blotting

Kidney samples stored in liquid were ultrasonicated at 4°C in the presence of phosphatase inhibitor and protease inhibitor and then centrifuged at 14000 rpm for 15 min. The supernatants were collected, and protein concentrations were determined using a BCA protein assay kit. Protein samples were separated by SDS-PAGE and transferred to polyvinylidene fluoride membranes. The membrane was blocked using 5% bovine serum albumin for 2 h, incubated with a primary antibody against TGF-*β*1 (1 : 1000) and glyceraldehyde 3-phosphate dehydrogenase (GAPDH; 1 : 5000) at 4°C overnight. After washing three times in Tris-buffered saline containing Tween 20 (TBST), each membrane was incubated with horseradish peroxidase- (HRP-) conjugated IgG (1 : 10000) at room temperature for 2 h. Protein bands were detected using enhanced chemiluminescence, and band intensities were determined as compared with that of GAPDH.

### 2.10. Statistical Analysis

Data are expressed as $\bar{x}\pm s$ and were analyzed using SPSS 25.0 (SPSS Inc., USA). Differences among the three groups were analyzed using ANOVA followed by least significant difference *t*-test. Differences between two groups were analyzed using Student's *t*-test. Values of *p* < 0.05 were considered statistically significant.

## 3. Results

### 3.1. mRNA and Protein Expression of TGF-*β*1, miR-130b Expression, and Renal Pathological and Fibrogenic Changes Were Compared with Baseline Group

#### 3.1.1. Expression of TGF-*β*1

**Figure 1:**
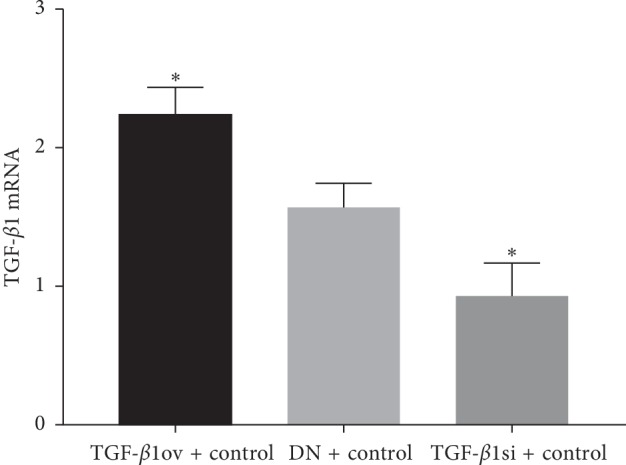
mRNA expression of TGF-*β*1. TGF-*β*1ov + control, TGF-*β*1 overexpression + oil; TGF-*β*1si + control, TGF-*β*1 siRNA + oil; DN + control, DN + oil. ^*∗*^*p* < 0.05 vs DN + control.

**Figure 2:**
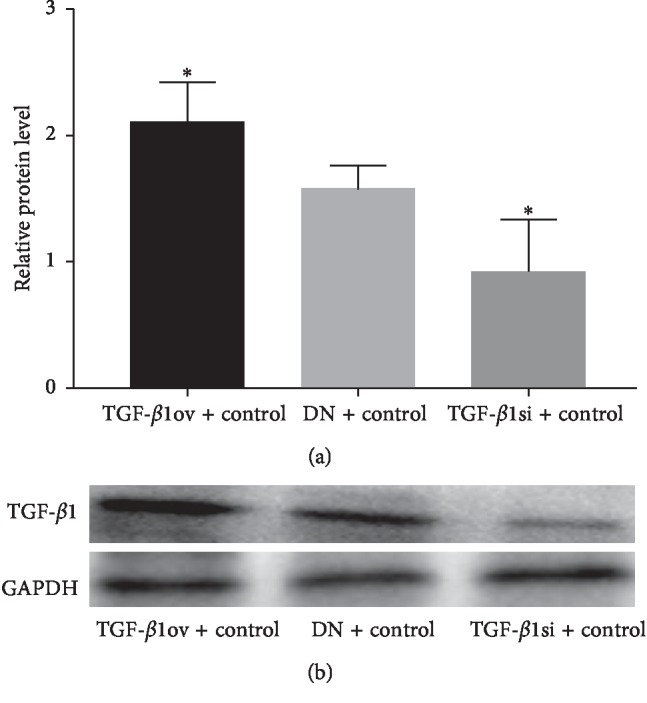
Protein expression of TGF-*β*1. (a) Relative protein. (b) Western blotting. TGF-*β*1ov + control, TGF-*β*1 overexpression + oil; TGF-*β*1si + control, TGF-*β*1 siRNA + oil; DN + control, DN + oil. ^*∗*^*p* < 0.05 vs DN + control.

The mRNA and protein expression of TGF-*β*1 was significantly higher in the TGF-*β*1 overexpression + oil group than in the DN + oil group (*p* < 0.05). By contrast, the mRNA and protein expression of TGF-*β*1 was significantly lower in the TGF-*β*1 siRNA + oil group than in the DN + oil group (*p* < 0.05). These data indicated the successful establishment of DN in rats expressing different levels of TGF-*β*1 (Table 2 and Figures 1, 2(a), and 2(b)).

#### 3.1.2. Renal Histological and Fibrogenic Changes

**Figure 3:**
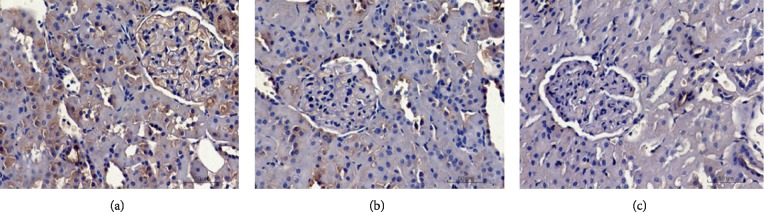
Detection of TGF-*β*1 by immunohistochemistry in TGF-*β*1 overexpression + oil (a), DN + oil (b), and TGF-*β*1 siRNA + oil (c) groups.

*(1) Immunohistochemistry*. Immunohistochemistry analysis (Figure 3) revealed that TGF-*β*1 expression was significantly higher in the TGF-*β*1 overexpression + oil group compared to that in the DN + oil group (*p* < 0.05). By contrast, TGF-*β*1 was significantly lower in the TGF-*β*1 siRNA + oil group compared to that in the DN + oil group (*p* < 0.05). These data suggested the successful establishment of DN rats expressing different levels of TGF-*β*1.

**Figure 4:**
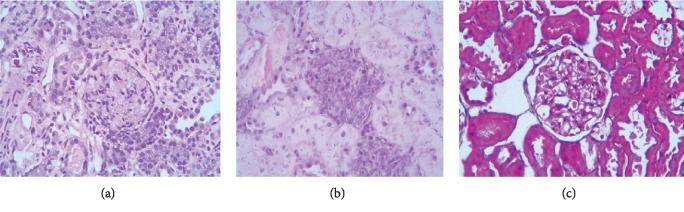
HE staining revealed renal pathological changes in DN rats in the TGF-*β*1 overexpression + oil (a), DN + oil (b), and TGF-*β*1 siRNA + oil (c) groups.

*(2) HE Staining*. HE staining (Figure 4) revealed glomeruli with an increased diameter and cell number in the TGF-*β*1 overexpression + oil group. Disappearance of glomerular Bowman's capsule, Bowman's cystic wall, and visceral adhesions was evident, as was severe renal tubular edema with partial necrosis. In the DN + oil group, regular glomeruli, shrunken Bowman's capsule, and moderately edematous renal tubules were evident. In the TGF-*β*1 siRNA + oil group, regular glomeruli, recovered Bowman's capsule, and renal tubules with slight edema were evident.

**Figure 5:**
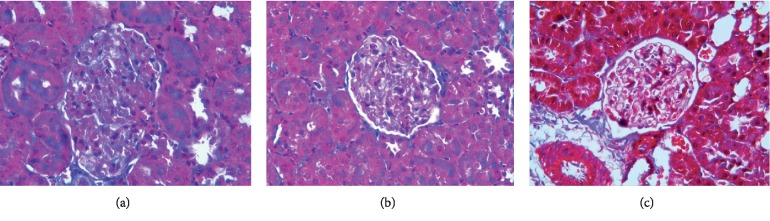
Masson staining revealing fibrogenic changes in terms of renal fibrosis in DN rats in the TGF-*β*1 overexpression + oil (a), DN + oil (b), and TGF-*β*1 siRNA + oil (c) groups.

*(3) Masson Staining*. Masson staining (Figure 5) revealed a large amount of blue-stained collagen fibers and little red-stained normal tissues in kidney tissue from rats in the TGF-*β*1 overexpression + oil group. In the DN + oil group, the kidney tissues showed local collagen fiber deposition. By contrast, in the TGF-*β*1 siRNA + oil group, only a small amount of collagen fiber deposition was observed.

#### 3.1.3. Expression of miR-130b

**Figure 6:**
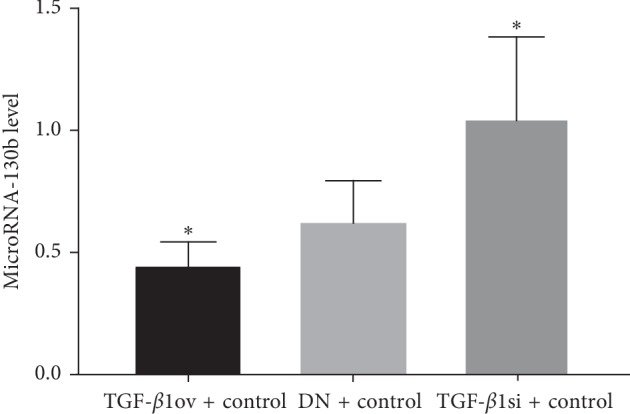
MicroRNA-130b expression. TGF-*β*1ov + control, TGF-*β*1 overexpression + oil; TGF-*β*1si + control, TGF-*β*1 siRNA + oil; DN + control, DN + oil. ^*∗*^*p* < 0.05 vs DN + control.

The expression of miR-130b was significantly lower in the TGF-*β*1 overexpression + oil group than that in the DN + oil group (*p* < 0.05). Conversely, miR-130b expression was obviously increased in the TGF-*β*1 siRNA + oil group compared to the DN + oil group (*p* < 0.05) (Table 3, Figure 6).

### 3.2. mRNA and Protein Expression of TGF-*β*1, miR-130b Expression, and Renal Pathological and Fibrogenic Changes Were Compared with Model Groups

#### 3.2.1. Expression of TGF-*β*1

**Figure 7:**
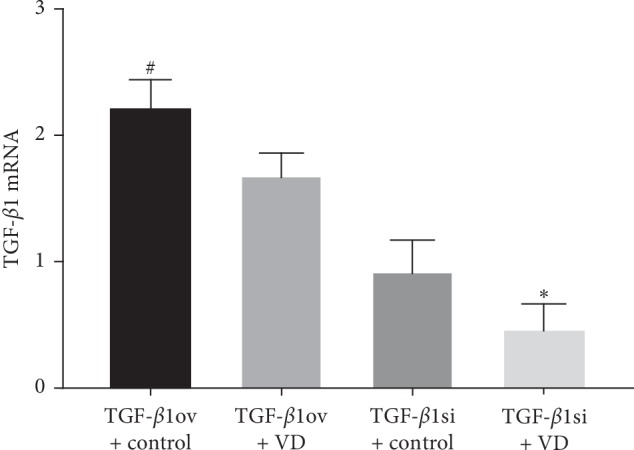
TGF-*β*1 mRNA expression. TGF-*β*1ov + control, TGF-*β*1 overexpression + oil; TGF-*β*1 ov + VD, TGF-*β*1 overexpression + 1,25(OH)2D3; TGF-*β*1si + control, TGF-*β*1 siRNA + oil; TGF-*β*1si + VD, TGF-*β*1 siRNA + 1,25(OH)2D3. ^*∗*^*p* < 0.05 vs TGF-*β*1si + control; ^#^*p* < 0.05 vs TGF-*β*1ov + VD.

**Figure 8:**
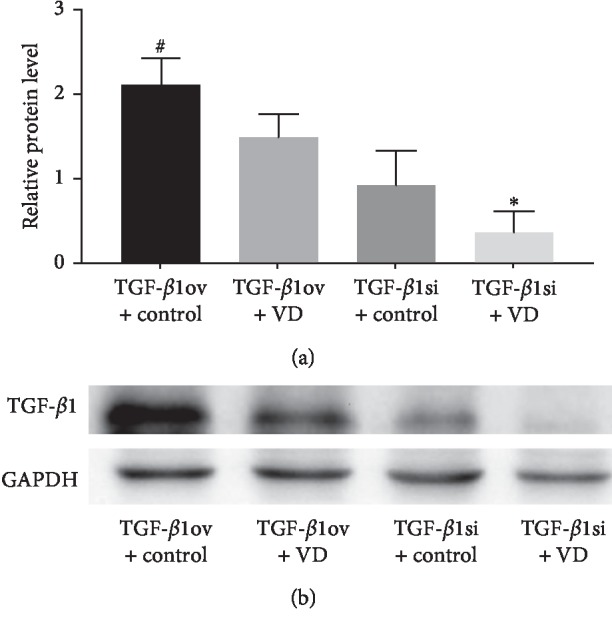
TGF-*β*1 protein expression. (a) Relative protein level and (b) western blotting. TGF-*β*1ov + control, TGF-*β*1 overexpression + oil; TGF-*β*1 ov + VD, TGF-*β*1 overexpression +1,25(OH)2D3; TGF-*β*1si + control, TGF-*β*1 siRNA + oil; TGF-*β*1si + VD, TGF-*β*1 siRNA + 1,25(OH)2D3. ^*∗*^*p* < 0.05 vs TGF-*β*1si + control; ^#^*p* < 0.05 vs TGF-*β*1ov + VD.

TGF-*β*1 mRNA and protein levels were decreased in the TGF-*β*1 overexpression + 1,25(OH)2D3 group compared to the TGF-*β*1 overexpression + oil group (*p* < 0.05). The mRNA and protein expression of TGF-*β*1 was significantly lower in the TGF-*β*1 siRNA + 1,25(OH)2D3 group than that in the TGF-*β*1 siRNA + oil group (*p* < 0.05) (Table 4 and Figures 7, 8(a) and 8(b)).

#### 3.2.2. Renal Pathological and Fibrogenic Changes

**Figure 9:**
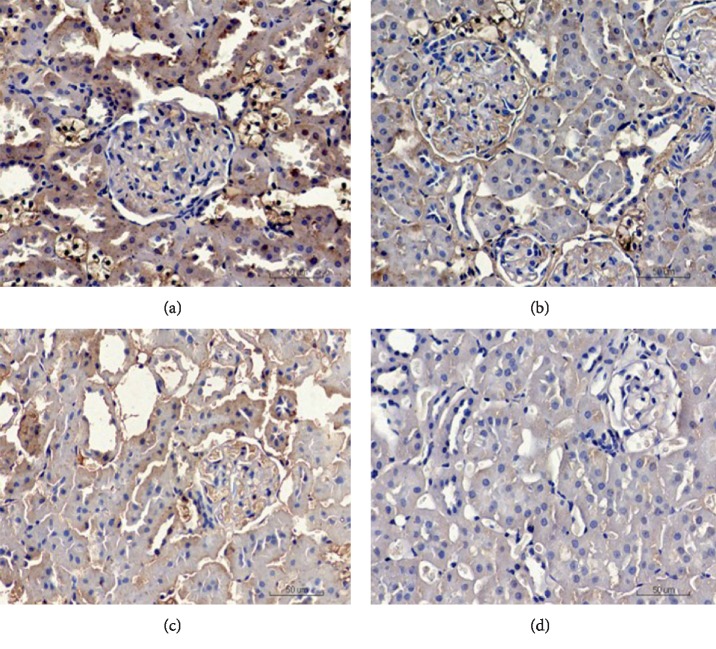
TGF-*β*1 expression detected by immunohistochemistry in the TGF-*β*1 overexpression + oil (a), TGF-*β*1 overexpression + 1,25(OH)2D3 (b), TGF-*β*1 siRNA + oil (c), and TGF-*β*1 siRNA + 1,25(OH)2D3 (d) groups.

*(1) Immunohistochemistry*. Immunohistochemistry analysis (Figure 9) showed that the TGF-*β*1 mRNA and protein levels were significantly lower in the TGF-*β*1 overexpression + 1,25(OH)2D3 group those in the TGF-*β*1 overexpression + oil group. TGF-*β*1 mRNA and protein expression levels were also decreased in the TGF-*β*1 siRNA + 1,25(OH)2D3 group compared to the TGF-*β*1 siRNA + oil group.

**Figure 10:**
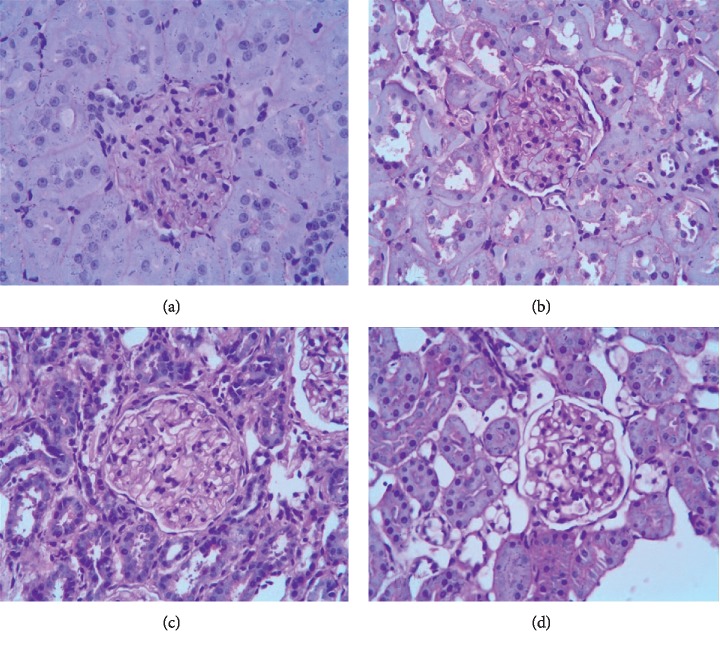
HE staining revealing the renal histological changes in DN rats in the TGF-*β*1 overexpression + oil (a), TGF-*β*1 overexpression + 1,25(OH)2D3 (b), TGF-*β*1 siRNA + oil (c), and TGF-*β*1 siRNA + 1,25(OH)2D3 (d) groups.

*(2) HE Staining*. HE staining (Figure 10) revealed irregular glomeruli with increased diameter and cell number in the TGF-*β*1 overexpression + oil group. Disappearance of glomerular Bowman's capsule, Bowman's cystic wall, and visceral adhesions was evident, as well as severe renal tubular edema with partial necrosis and inflammatory cell infiltration in renal interstitium. In the TGF-*β*1 overexpression + 1,25(OH)2D3 group, regular glomeruli, shrunken Bowman's capsule, and moderately edematous renal tubules were evident. In TGF-*β*1 siRNA + oil group, regular glomeruli, shrunken Bowman's capsule, and renal tubules with moderate edema were evident; however, there was also slight inflammatory cell infiltration in the renal interstitium. In the TGF-*β*1 siRNA + 1,25(OH)2D3 group, regular glomeruli, recovered Bowman's capsule, and renal tubules with slight edema were observed.

**Figure 11:**
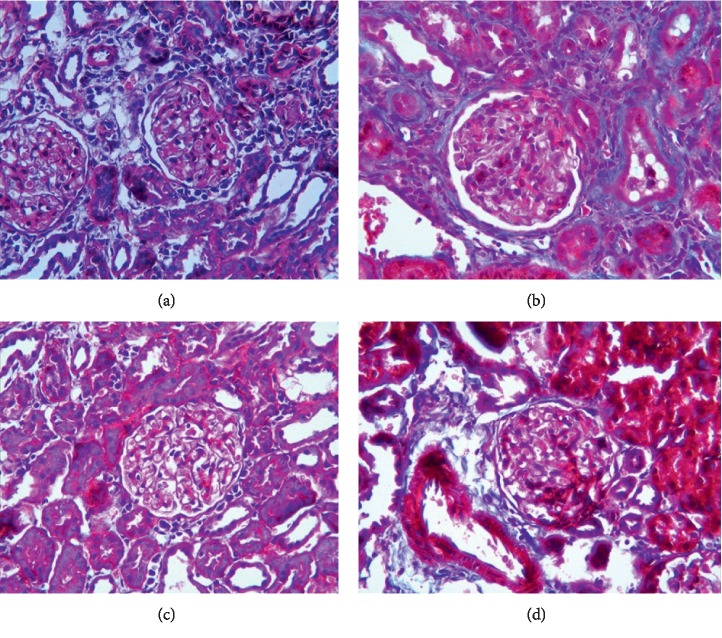
Masson stain revealing renal fibrosis in DN rats in the TGF-*β*1 overexpression + oil (a), TGF-*β*1 overexpression + 1,25(OH)2D3 (b), TGF-*β*1 siRNA + oil (c), and TGF-*β*1 siRNA + 1,25(OH)2D3 (d) groups.

*(3) Masson Staining*. Masson staining (Figure 11) revealed kidney tissue with a large amount of blue-stained collagen fibers and some red-stained normal tissues in the TGF-*β*1 overexpressing + oil group. In the TGF-*β*1 overexpression + 1,25(OH)2D3 group, the kidney tissues showed local collagen fiber deposition. In the TGF-*β*1 siRNA + oil group, partial collagen fiber deposition was observed. In the TGF-*β*1 siRNA + 1,25(OH)2D3 group, only a small amount of collagen fiber deposition was detected.

#### 3.2.3. Expression of miR-130b

**Figure 12:**
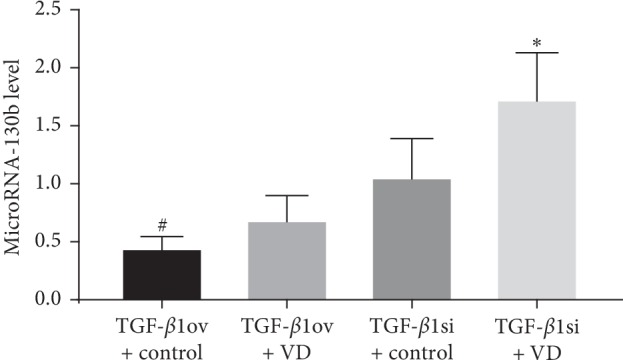
MiR-130b expression levels in DN rats. TGF-*β*1ov + control, TGF-*β*1 overexpression + oil; TGF-*β*1 ov + VD, TGF-*β*1 overexpression + 1,25(OH)2D3; TGF-*β*1si + control, TGF-*β*1 siRNA + oil; TGF-*β*1si + VD, TGF-*β*1 siRNA + 1,25(OH)2D3. ^*∗*^*p* < 0.05 vs TGF-*β*1si + control; ^#^*p* < 0.05 vs TGF-*β*1ov + VD.

After treatment with 1,25(OH)2D3, miR-130b expression was significantly higher in the TGF-*β*1 overexpression group than in the oil group (*p* < 0.05). Similarly, miR-130b was also elevated in the TGF-*β*1 siRNA + 1,25(OH)2D3 group compared to the TGF-*β*1 siRNA + oil group (*p* < 0.05) (Table 5 and Figure 12).

## 4. Discussion

In the present study, two DN rat models were established using lentivirus-mediated overexpression and downregulation TGF-*β*1 in kidney tissue. The role of miR-130b in the 1,25(OH)2D3-mediated improvement of renal fibrosis via TGF-*β*1 was assessed. The miR-130 family is associated with cardiac, lung, and renal fibrosis. Of note, miR-130b alleviates lung fibrosis by regulating collagen I and insulin-like growth factor1 expression [23, 24]. Moreover, miR-130b-3p is involved in renal damage in early lupus nephritis [25]. In type II diabetic patients, miR-130b is obviously reduced [26, 27] and affects endothelial cell function [28]. A critical role of miR-130b in renal tubule interstitial fibrosis in DN has been reported. Decreased serum miR-130b levels have been associated with declined renal function in DN patients. Moreover, miR-130b reportedly alleviates renal fibrosis via nail signals and collagen IV in high-glucose cultured NRK-52E cells and streptozotocin-induced DN rats [29]. A recent study showed that TGF-*β* may affect the expression of miR-130b, leading to renal fibrosis [30]. Upregulation of TGF-*β*1 may lead to downregulation of miR-130b and aggravate renal fibrosis in DN [17].

In this study, we investigated the changes in miR-130b and renal fibrosis in DN rats with different expression levels of TGF-*β*1 and explored whether miR-130b was regulated by TGF-*β*1 in the kidneys of these rats. The TGF-*β*1 mRNA and protein expression was significantly lower in the TGF-*β*1 siRNA + oil group of DN rats compared to the TGF-*β*1 overexpression + oil group. Moreover, HE and Masson staining showed that, compared to the TGF-*β*1 overexpression + oil group, rats in the TGF-*β*1 siRNA + oil group displayed reduced collagen fiber deposition, alleviated renal fibrosis, and elevated miR-130b expression. These results suggest that miR-130b may be negatively regulated by TGF-*β*1 and play a beneficial role in renal fibrosis in DN rats. The underlying mechanism is still unclear. Studies have shown that 1,25(OH)2D3 beneficially affects renal fibrosis in DN via TGF-*β*1, signal transducer and activator of transcription 1, protein tyrosine phosphatase nonreceptor type 2, and Nrf2 [31–33]. Studies in animal models show that 1,25(OH)2D3 can inhibit TGF-*β*1 and improve renal fibrosis in diabetic rats [5]. In addition, oral cholecalciferol obviously decreases albuminuria and urinary TGF-*β*1 in patients with type 2 DN by inhibiting the renin-angiotensin-aldosterone system [34]. In our study, TGF-*β*1 was negatively regulated by miR-130b. We further explored whether miR-130b was involved in the 1,25(OH)2D3-mediated improvement of renal fibrosis via TGF-*β*1. TGF-*β*1 mRNA and protein expression was decreased in the TGF-*β*1 siRNA + 1,25(OH)2D3 group. Moreover, HE and Masson staining showed that 1,25(OH)2D3 treatment significantly improved the damage to glomeruli and tubular structure as well as renal fibrosis and promoted the expression of miR-130b. Meanwhile, in TGF-*β*1 overexpression rats, 1,25(OH)2D3 treatment decreased the mRNA and protein expression of TGF-*β*1. HE and Masson staining in these rats also showed that 1,25(OH)2D3 treatment significantly improved the damage to glomeruli and tubular structure and renal fibrosis and promoted the expression of miR-130b. These data indicate the involvement of miR-130b in the 1,25(OH)2D3-mediated improvement of renal fibrosis in DN via TGF-*β*1 and the negative regulation of miR-130b by TGF-*β*1, which serves as a protective factor for DN. The precise mechanism through which TGF-*β* regulates miR-130b in 1,25(OH)2D3-treated DN is still unclear.

## 5. Conclusion

TGF-*β*1 negatively regulates miR-130b during the progression of DN, and miR-130b may be involved in the 1,25(OH)2D3-mediated improvement of DN via TGF-*β*1. Therefore, miR-130b may be developed as a novel therapeutic target for DN in the future. In addition, these findings provide a theoretical and experimental basis for the beneficial role of 1,25(OH)2D3 in DN.

## Figures and Tables

**Table 1:** Gene sequences of primers.

Gene	Forward primer	Reverse primer
TGF-*β*1	5′-GGCACCATCCATGACATGAACCG-3′	5′-GCCGTACACAGCACTTCTTCTCTG-3′
*β*-actin	5′-CAACCTTCTTGCAGCTCCTC-3′	5′-CGGTGTCCCTTCTGAGTGTT-3′
miR-130b	5′-ACUCUUUCCCUGUUGCACUAC-3′	5′-CAGUGCAAUGAUGAAGGGCAU-3′

TGF-*β*1, transforming growth factor β1; F, forward primer; R, reverse primer.

**Table 2:** TGF-*β*1 mRNA and protein expression.

Groups	*n*	mRNA	Protein
		TGF-*β*1	TGF-*β*1
TGF-*β*1ov + control	6	2.24 ± 0.20	2.11 ± 0.31
DN + control	6	1.57 ± 0.17	1.58 ± 0.18
*t*		6.252	3.59
*p*		<0.01	<0.01
TGF-*β*1si + control	6	0.93 ± 0.24	0.92 ± 0.41
DN + control	6	1.57 ± 0.17	1.58 ± 0.18
*t*		−5.33	−3.37
*p*		<0.01	<0.01

Data are expressed as mean ± standard deviation. TGF-*β*1ov + control, TGF-*β*1 overexpression + oil; TGF-*β*1si + control, TGF-*β*1 siRNA + oil; DN + control, DN + oil.

**Table 3:** MicroRNA-130b expression.

Groups	*n*	MicroRNA-130b
TGF-*β*1ov + control	6	0.46 ± 0.09
DN + control	6	0.64 ± 0.16
*t*		2.91
*p*		<0.05
TGF-*β*1si + control	6	1.07 ± 0.32
DN + control	6	0.64 ± 0.16
*t*		−2.47
*p*		<0.05

Data are expressed as mean ± standard deviation. TGF-*β*1ov + control, TGF-*β*1 overexpression + oil; TGF-*β*1si + control, TGF-*β*1 siRNA + oil; DN + control, DN + oil.

**Table 4:** TGF-*β*1 mRNA and protein expression.

Groups	*n*	mRNA	Protein
		TGF-*β*1	TGF-*β*1
TGF-*β*1ov + control	6	2.24 ± 0.20	2.11 ± 0.31
TGF-*β*1ov + VD	6	1.70 ± 0.16	1.50 ± 0.27
*t*		5.08	3.54
*p*		<0.01	<0.01
TGF-*β*1si + control	6	0.93 ± 0.24	0.92 ± 0.41
TGF-*β*1si + VD	6	0.48 ± 0.19	0.37 ± 0.24
*t*		3.53	2.82
*p*		<0.01	<0.01

Data are expressed as mean ± standard deviation. TGF-*β*1ov + control, TGF-*β*1 overexpression + oil; TGF-*β*1 ov + VD, TGF-*β*1 overexpression + 1,25(OH)2D3; TGF-*β*1si + control, TGF-*β*1 siRNA + oil; TGF-*β*1si + VD, TGF-*β*1 siRNA + 1,25(OH)2D3.

**Table 5:** MiR-130b expression in DN rats.

Group	Number	MicroRNA-130b
TGF-*β*1ov + control	6	0.46 ± 0.09
TGF-*β*1ov + VD	6	0.70 ± 0.20
*t*		2.64
*p*		<0.05
TGF-*β*1si + control	6	1.07 ± 0.32
TGF-*β*1si + VD	6	1.74 ± 0.39
*t*		3.19
*p*		<0.05

Data are expressed as mean ± standard deviation. TGF-*β*1ov + control, TGF-*β*1 overexpression + oil; TGF-*β*1 ov + VD, TGF-*β*1 overexpression + 1,25(OH)2D3; TGF-*β*1si + control, TGF-*β*1 siRNA + oil; TGF-*β*1si + VD, TGF-*β*1siRNA + 1,25(OH)2D3.

## Data Availability

The data analyzed during the study are not publicly available. Rigorous analysis of the data in order to ensure the objective authenticity of the results was done.
